# Mitochondrial orchestration of PANoptosis: mechanisms, disease pathogenesis, and emerging therapeutic frontiers

**DOI:** 10.1038/s41420-025-02750-z

**Published:** 2025-10-21

**Authors:** Jiale Zhang, Xiaoya Fu, Feifan Jia, Yahao Jing, Jiameng Zhang, Aoxue Bai, Mengyuan Cui, Lei Zhang, Kunhou Yao

**Affiliations:** 1 Department of General Surgery, the Second Clinical Medical College of Henan University (Huaihe Hospital), Henan University, Kaifeng, Henan China; 2 School of Basic Medical Sciences, Henan University, Kaifeng, Henan China

**Keywords:** Cell death, Diseases

## Abstract

Mitochondria, traditionally known as cellular powerhouses, are now recognized as central regulators of programmed cell death (PCD) and key players in disease pathogenesis. This review synthesizes current knowledge on mitochondrial mechanisms in PANoptosis—a convergent pathway integrating apoptosis, necroptosis, and pyroptosis—and their implications in diverse pathologies. Mitochondria govern intrinsic apoptosis via Bcl-2 family proteins and mitochondrial outer membrane permeabilization (MOMP), amplify necroptosis through RIPK1/RIPK3-driven ROS signaling, and indirectly regulate pyroptosis via inflammasome-mitochondria crosstalk. Dysfunctional mitochondria contribute to neurodegenerative diseases, cardiovascular disorders, cancer, and autoimmune/metabolic syndromes. Emerging therapies targeting mitochondrial pathways, such as Bcl-2 inhibitors, mPTP modulators, and ROS-inducing agents, demonstrate clinical promise in restoring cell death sensitivity and mitigating inflammation. By bridging molecular mechanisms with therapeutic innovations, this work underscores mitochondria as dynamic hubs of cellular fate and disease intervention.

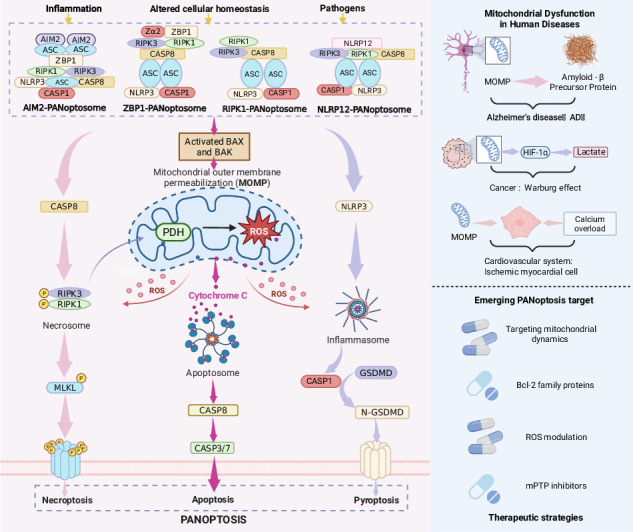

## Facts

- Mitochondria act as central regulators of PANoptosis by coordinating apoptosis, necroptosis, and pyroptosis into a unified cell death network.
- Mitochondrial dysfunction drives pathogenesis in neurodegeneration, cancer, and autoimmune diseases by disrupting death-inflammation balance.
- Targeting mitochondrial pathways restores cell death sensitivity and suppresses inflammation in preclinical models.
- Cancer cells exploit mitochondrial metabolic plasticity to evade apoptosis, while neuronal/cardiac cells succumb to mitochondrial ROS and calcium overload.

## Open questions

- How do mitochondria spatially and temporally prioritize apoptosis, necroptosis, or pyroptosis during PANoptosome assembly under distinct stressors?
- Can mitochondrial OXPHOS suppression in cancer be selectively targeted to amplify BH3 mimetic efficacy without harming normal cells?
- Does mtDNA leakage universally activate NLRP3/MAVS across tissues, or are organ-specific mitochondrial sensors involved?
- How to modulate mitochondrial dynamics or mitophagy to inhibit PANoptosis-driven pathology while preserving homeostasis?

## Introduction

Traditionally regarded as the cell’s “powerhouse” due to its central role in ATP production, mitochondria have evolved into key regulators of cell fate through their involvement in PCD. Beyond energy metabolism, mitochondria orchestrate complex signaling networks governing apoptosis, necroptosis, and pyroptosis—three distinct yet interconnected modes of PCD regulated by mitochondria [1, 2]. Recent studies have unveiled a novel integrated cell death pathway termed PANoptosis, which unifies these pathways into a cohesive framework, highlighting mitochondria as a crucial nexus for cellular stress responses and death decisions [3].

Apoptosis, a non-inflammatory form of PCD, is tightly regulated by mitochondrial dynamics. The intrinsic pathway is mediated by Bcl-2 family proteins and relies on MOMP to trigger Cyt crelease and caspase activation [4]. In contrast, necroptosis is a non-caspase-dependent inflammatory cell death that depends on the RIPK1/RIPK3/MLKL signaling pathway but can be amplified by mtROS and metabolic reprogramming [5]. Pyroptosis is executed by gasdermin D (GSDMD) pores and indirectly involves mitochondria through MOMP induction and inflammasome interactions [6]. These pathways converge in PANoptosis, where mitochondria serve as sensors and amplifiers of death signals, integrating stress signals through mitochondrial DNA leakage, ROS signaling, and dynamic membrane remodeling.

Mitochondrial dysfunction is implicated in a spectrum of diseases, including neurodegenerative disorders, cardiovascular diseases, cancer, and metabolic syndrome [7]. For instance, in Alzheimer’s disease, mitochondrial calcium overload and oxidative stress drive neuronal apoptosis and β-amyloid aggregation [8]. In cancer, metabolic reprogramming and overexpression of anti-apoptotic Bcl-2 confer tumor survival and chemoresistance [9]. Therapeutic strategies targeting mitochondrial pathways, such as Bcl-2 inhibitors, mPTP modulators, and ROS inducers, are revolutionizing disease management by restoring apoptotic sensitivity or attenuating inflammatory cascades [10–12].

This review systematically explores the dual role of mitochondria in cell life-and-death decisions and disease pathogenesis. We elucidate their regulatory mechanisms in PANoptosis, dissect their roles in major human diseases, and evaluate emerging therapeutic interventions targeting mitochondrial pathways. By bridging molecular insights with clinical applications, this work underscores the dynamic nature of mitochondria as vital organelles for both health and disease, paving the way for novel targeted therapies.

## PANoptosis: a mitochondrially orchestrated integrated cell death mechanism

PANoptosis is an integrated mechanism of programmed cell death that involves the interaction of multiple modes of cell death, including apoptosis, necroptosis, and pyroptosis role in this process [13].

### Mitochondria as a central hub for PANoptosis cross-talk

Although the mechanisms of mitochondria in apoptosis, necroptosis, and pyroptosis have been detailed separately (see Sections “Mitochondria and pyroptosis”, “Mitochondria and apoptosis”, and “Mitochondria and programmed necrosis”), the key to PANoptosis lies in the high degree of crosstalk and synergy among these three death pathways, with mitochondria at the center of this interactive network. MOMP, as a shared execution node, not only mediates cytochrome *c* release and apoptotic vesicle formation, but also amplifies pyroptotic and necroptotic signals through the release of DAMPs—such as mitochondrial DNA (mtDNA), mtROS, and cardiolipin—and activation of NLRP3 inflammasomes [14–17]. The N-terminal fragment of GSDMD-NT disrupts the plasma membrane while also directly attacking mitochondrial membranes, leading to mtROS release, decreased membrane potential, and impaired oxidative phosphorylation (OXPHOS). This induces the efflux of mitochondrial proteins and mtDNA, further activating MOMP and creating a feedback loop that drives caspase-dependent apoptosis [18].

At the level of pathway regulation, the balance of caspase signaling is also regulated by mitochondrial integrity. For example, the inhibition of caspase-8 promotes necroptosis, whereas the activation of caspase-1, -4, -5, and -11 drives scorched-earth death [19–21]. Thus, mitochondrial dysfunction is not only the result of individual death pathways but also actively shapes the dynamic equilibrium of multiple pathway linkages in PANoptosis through the release of common molecular triggers, shared execution mechanisms, and metabolic signals.

### Mitochondria and pyroptosis

**Fig. 1:**
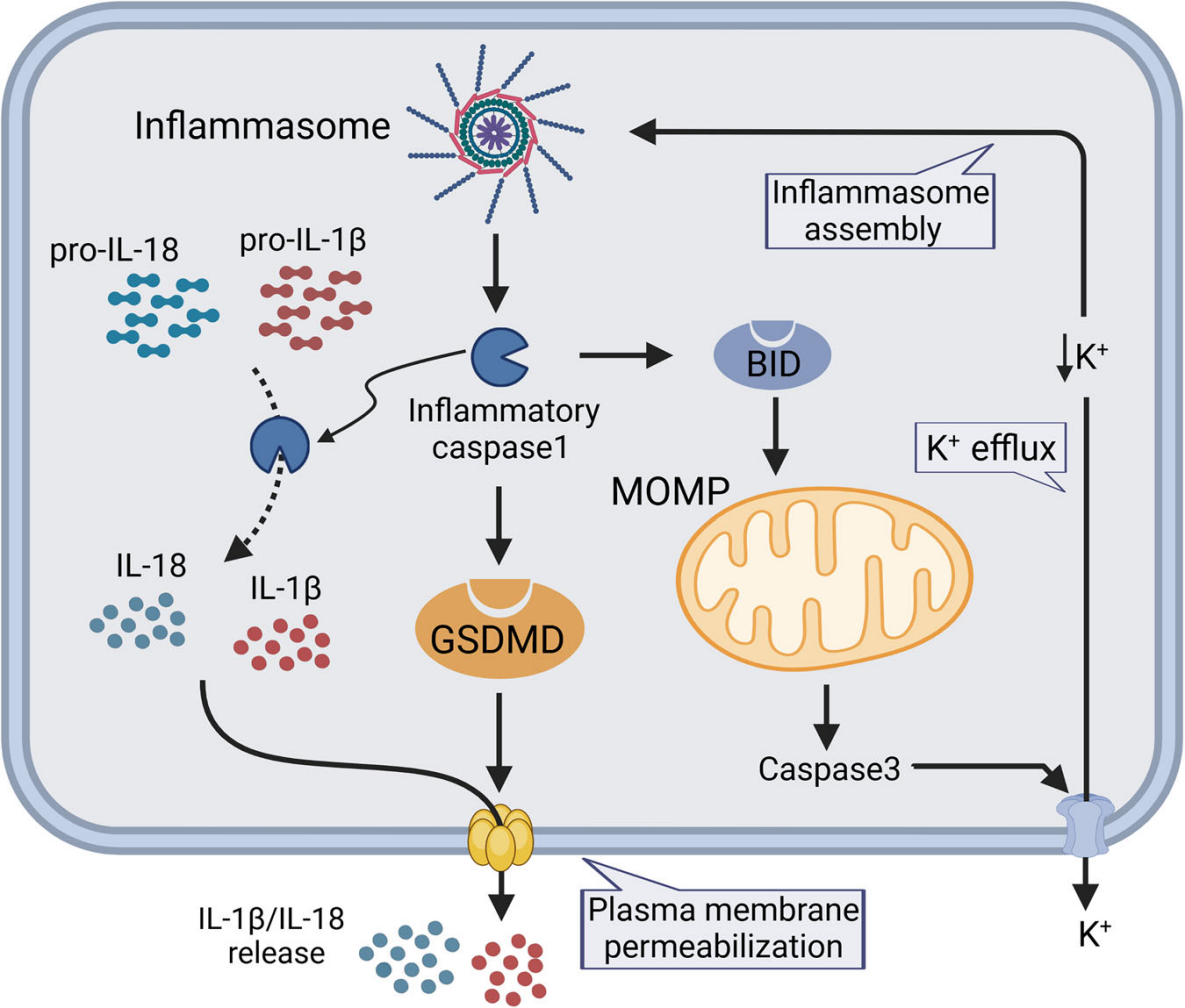
The link between mitochondria and pyroptosis. The presence of certain intracellular pathogens activates inflammatory caspases, which subsequently activate pro-inflammatory cytokines IL-1β and IL-18. Inflammatory caspase-1 also clears and activates GSDMD, which forms pores in the plasma membrane and penetrates, leading to pyroptosis. Active GSDMD also induced MOMP. In addition, the inflammasome activity cleans and activates the protein BID to induce MOMP. Subsequently, caspase-3 is activated and cleans the glycoprotein pannexin 1, leading to the formation of potassium channels on the plasma membrane and the efflux of potassium ions, which further promotes the formation of the inflammasome.

Pyroptosis, as a pro-inflammatory form of programmed cell death, is executed by cysteine asparaginase-dependent cleavage of gasdermin D, a mode of cell death that is regulated by the assembly of a multiprotein signaling complex called inflammasome by distinctive morphological manifestations such as cell swelling, DNA fragmentation, and plasma membrane rupture Inflammatory caspases also cleave and activate GSDMD [18, 22–24]. Mitochondria become dysfunctional before the GSDMD-dependent cell membrane ruptures are extensive interactions between cellular pyroptosis and mitochondrial apoptosis [25] (Fig. 1). First, as previously described, GSDMD amino-terminal cleavage fragments produced by inflammatory vesicles target not only the plasma membrane, but also specifically localize to the mitochondrial outer membrane, directly inducing MOMP through oligomerization, which leads to activation of 3 Cyst asparaginase cleaves dependently a glycoprotein forming potassium channels—pannexin 1, and activation of this protein leads to intracellular potassium efflux, which further promotes the assembly of inflammatory vesicles [26, 27]. Second, in cells expressing a small amount of GSDMD, activation of cysteinyl aspartate triggers mitochondrial apoptosis rather than cellular pyroptosis, and inflammatory vesicle-activated caspase-1 generates tBID by cleavage of BID, which synergistically forms MPTP with BAX/ a process negatively regulated by the MCL-1 protein [28, 29]. MOMP leads to a massive release of mtDNA and mtROS, signaling through the TLR9/STING pathway.

Caspase-1 plays a central role in pyroptosis, executing cell membrane rupture through activation of the inflammatory vesicle complex, cleavage of the pro-inflammatory cytokines IL-1β and IL-18, and cleavage of GSDMD, as well as linking the pyroptosis and mitochondrial apoptosis pathways through cleavage of BID proteins.

### Mitochondria and apoptosis

**Fig. 2:**
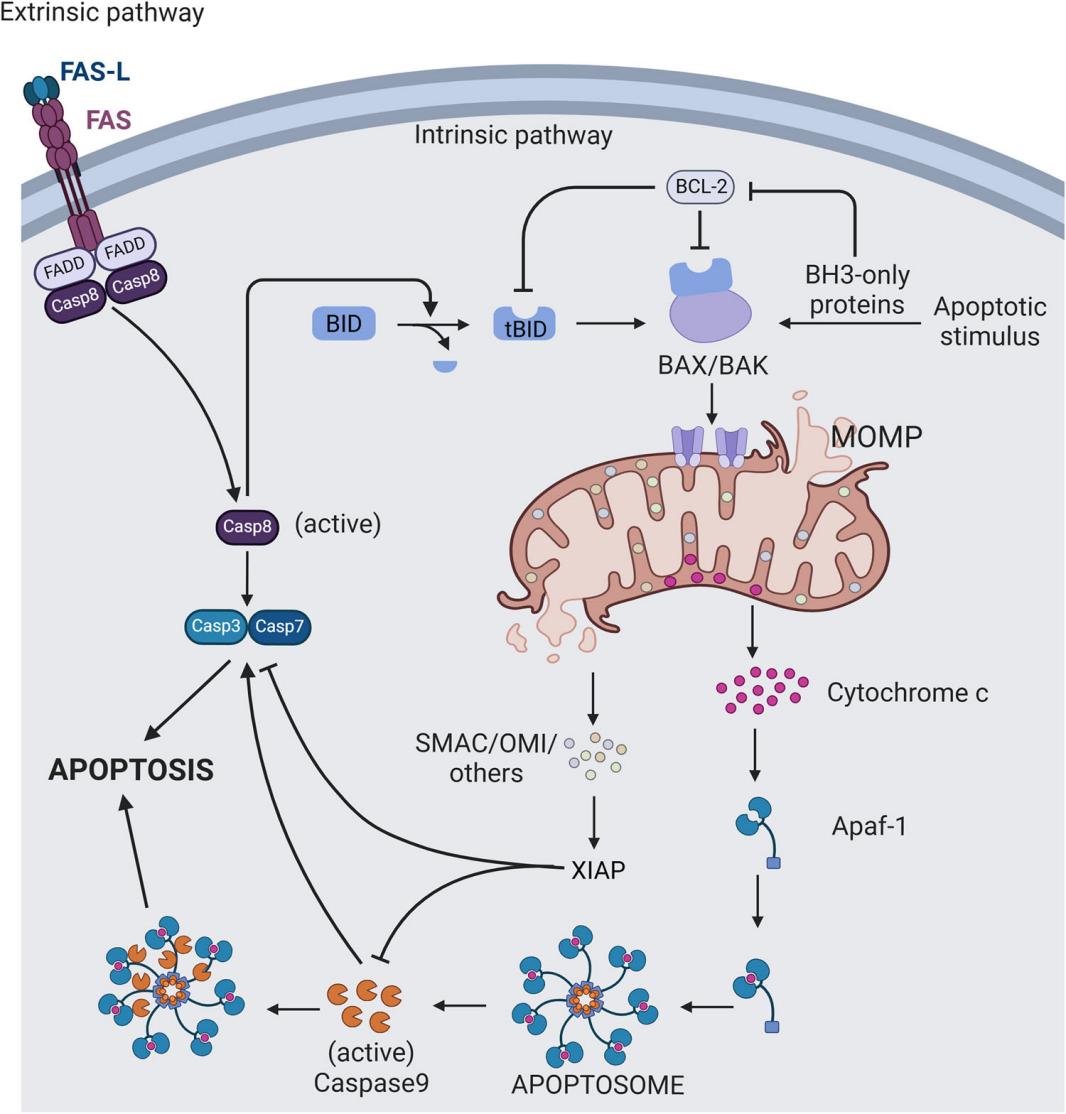
Apoptotic signaling pathways. The extrinsic apoptotic pathway is triggered by the binding of the death ligand Fas-L to the transmembrane receptor Fas, which activates caspase-8 and directly clears effector caspase-3/7 to trigger apoptosis execution. Meanwhile, caspase-8-mediated cleavage of BH3-only protein BID generates tBID, which translocate to the mitochondrial surface and cooperate with the pro-apoptotic proteins BAX/BAK to induce MOMP, release Cyt c, and form apoptotic bodies, thereby activating caspase-9-dependent endogenous apoptotic cascade.

Apoptosis is a highly regulated process of cell death that does not cause an inflammatory response. Apoptosis is characterized by affecting dispersed individual cells and is manifested histologically by cell shrinkage and fragmentation, nuclear cohesion and fragmentation, cytoplasmic cohesion, and the formation of apoptotic vesicles through two main pathways: exogenous and endogenous [30]. As the death receptor pathway, is initiated by the binding of a death ligand to its corresponding death receptor of Fas ligand to the Fas receptor, initiates apoptotic processes by recruiting Fas-associated death structural domain proteins (FADD) [31]. FADD further promotes the formation of the death-inducing signaling complex and dimerization of the articulin caspase-8, and triggers caspase-8 activation caspase-8 then cleaves and activates effector caspase-3 and caspase-7, triggering apoptotic hallmark events such as DNA breakage and cytoskeletal degradation endogenous apoptotic pathway, on the other hand, is mainly mediated by mitochondria pathway can be triggered by intracellular stress signals, including DNA damage, accumulation of ROS, withdrawal of growth factors, and mitotic termination, the dynamic equilibrium of the BCL-2 family of proteins [32]. MOMP also leads to the release of SMAC/DIABLO and OMI/HtrA2 from mitochondria, which inhibit the apoptosis inhibitory protein XIAP, relieving it from its inhibitory effect on caspases, thereby driving the apoptotic process caspase-8 cleavage of the BH3-only protein BID to generate the truncated tBID is a key link connecting the exogenous and endogenous apoptotic pathways [33] (Fig. 2). In addition, mitophagy exhibits a dual role in apoptosis—the PINK1/Parkin pathway removes MOMP-damaged mitochondria to limit apoptotic spreading, but under sustained stress, autophagy-associated protein caspase-dependent cleavage of ATG5 generates pro-apoptotic fragments, which in turn enhance mitochondrial localization of BAX and MOMP formation [34, 35].

Caspase-3, a key performer in the apoptotic process, is activated by caspase-9 and further cleaves a variety of substrates, leading to nuclear cohesion, DNA breaks, and cellular debris formation. This process is the final execution stage of apoptosis and ensures effective cell death.

### Mitochondria and programmed necrosis

**Fig. 3:**
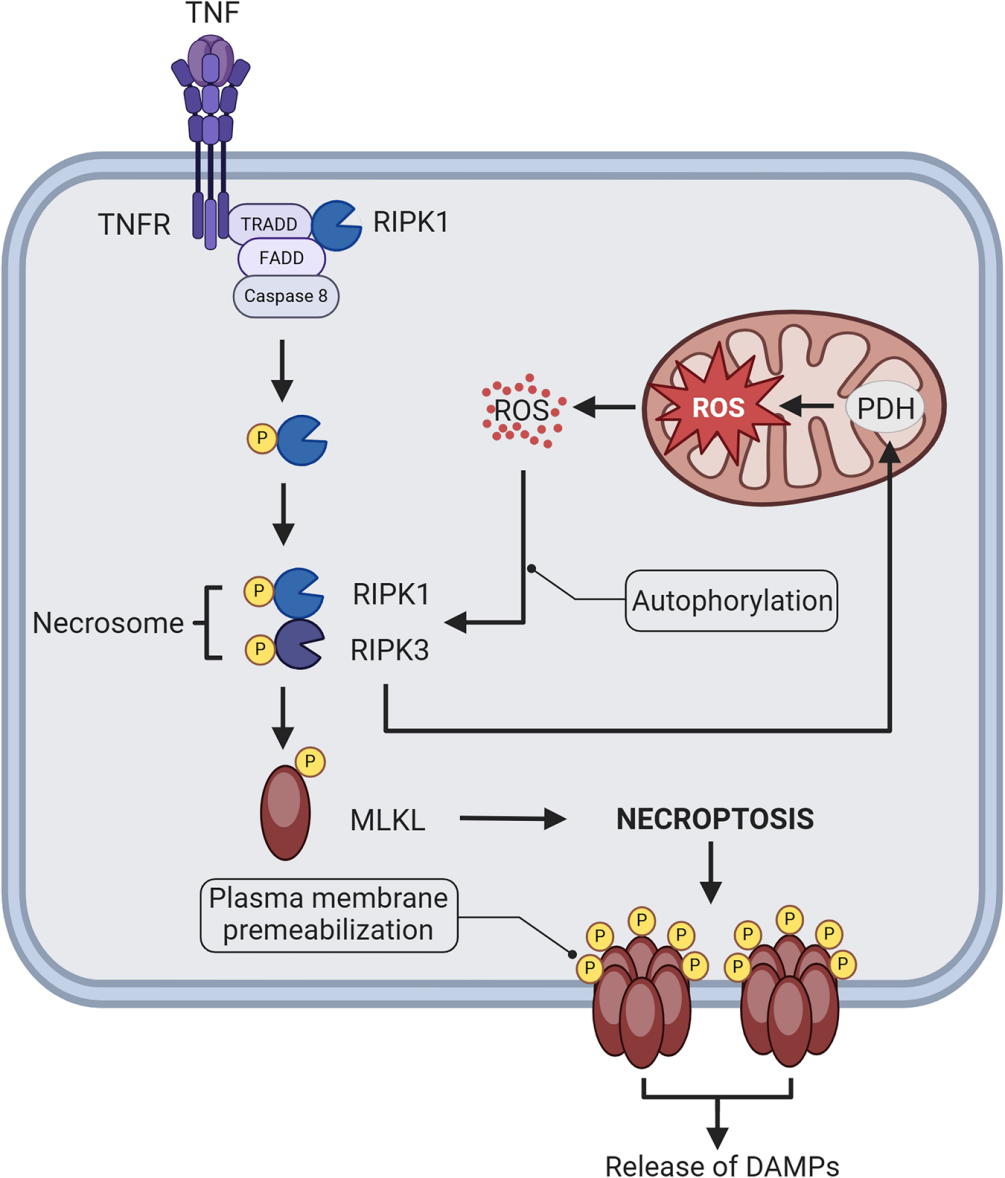
The necroptosis pathway and mitochondria. When caspase activity is inhibited, TNF receptor complexes form necrosomes through the RIPK1-RIPK3 kinase cascade, which drives MLKL phosphorylation and oligomerization, eventually leading to plasma membrane permeabilization and cell lysis. RIPK3 phosphorylates mitochondrial pyruvate dehydrogenase to produce superoxide anion and hydrogen peroxide (H₂O₂). Mitochondria-derived ROS modify RIPK1 at Cys257/Cys268 to enhance its kinase activity and promote necrosome assembly in a positive feedback manner.

Necrotic apoptosis is a pro-inflammatory pattern of cell death along with the release of DAMP, a regulated caspase-independent form morphology and inflammation common cell death features that are largely dependent on RIPK1 and RIPK3, with an unregulated passive cellular form called necrotic death apoptosis is characterized by programmed mitochondrial dysfunction and plasma membrane rupture induced by a variety of stimuli, including TNF-α, Toll-like receptor ligands, and chemotherapeutic agents [36–42]. Abnormal levels of necrotic apoptosis have been associated with a variety of inflammatory diseases and ischemic injury, making this mode of cell death an important therapeutic target [43, 44]. These receptors recruit the articulating proteins FADD, TRADD, and caspase-8 or caspase-10 to form an oligomeric complex, and active RIPK1 by the oligomeric complex [45]. The necrosome then phosphorylates and activates the pseudokinase mixed-spectrum kinase structural domain-like pseudokinase, which translocates to the plasma membrane and permeabilizes and opens up the calcium or sodium channels [46]. Past experiments involving mitochondrial depletion via autophagy or forced RIPK3 activation through chemically induced dimerization have demonstrated that necroptosis proceeds with identical kinetics regardless of mitochondrial presence. This supports MLKL activation as the execution mechanism of necroptosis [47]. However, recent studies reveal that mitochondria are not merely energy suppliers but also critical hubs for signal amplification. RIPK3 phosphorylates the mitochondrial pyruvate dehydrogenase complex and disrupts the inhibitory effect of pyruvate dehydrogenase kinase, driving oxidative decarboxylation of pyruvate to generate acetyl-CoA. This accelerates the tricarboxylic acid cycle and causes electron transport chain overload, resulting in a burst of superoxide anions (O₂^−^) and hydrogen peroxide (H₂O₂) [48]. Mitochondria-derived reactive oxygen species further amplify signaling by oxidatively modifying Cys257/Cys268 on RIPK1, enhancing its kinase activity and creating a self-sustaining “ROS-RIPK loop” that promotes necrosome assembly [49]. Notably, ROS levels may determine whether a cell initiates necroptosis. Progressive mitochondrial dysfunction—such as that observed during senescence—could heighten susceptibility to necroptotic cell death. The role of MLKL in necrotic apoptosis is to act as an executor, translocating to the plasma membrane and causing membrane permeabilization upon activation through its phosphorylation, leading to cell death (Fig. 3).

## Mitochondrial interactions with pan-apoptotic vesicles: common mechanisms and specific functions

**Fig. 4:**
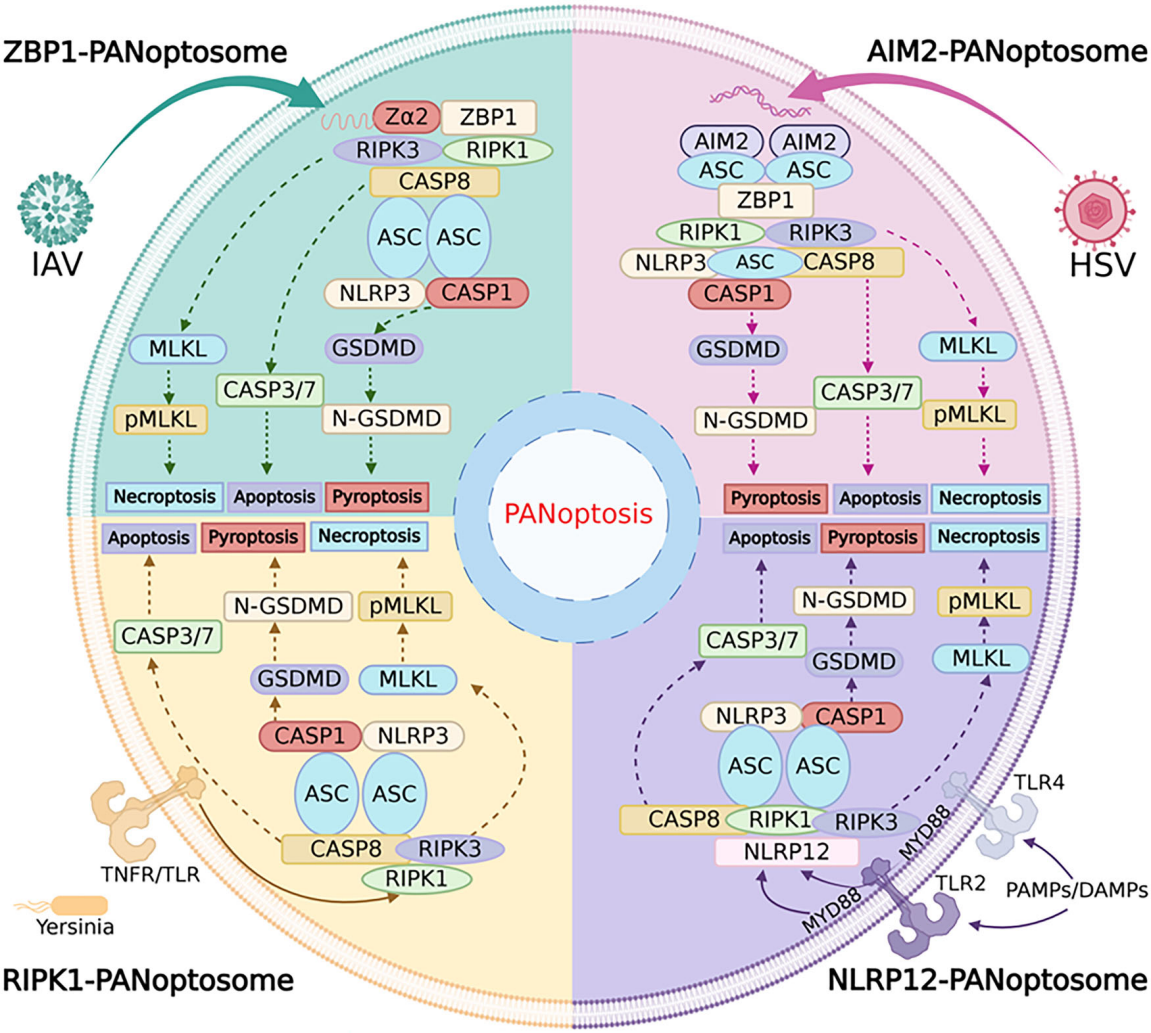
Regulatory mechanism of PANoptosis. Different pathogens activate specific PANoptosome complexes, which in turn trigger multiple cell death pathways. The upper left is ZBP1-PANoptosome activated by IAV, and the upper right is AIM2-PANoptosome activated by HSV. The lower left also shows Yersinia activating RIPK1-PANoptosome via TNFR/TLR, and the lower right activating NLRP12-PANoptosome via TLR2/4 and MyD88. These complexes interact through different signaling molecules, ultimately leading to multiple forms of cell death such as necroptosis, apoptosis, and pyroptosis.

PANoptosis represents an integrated inflammatory cell death mechanism that combines key features of apoptosis, necroptosis, and pyroptosis, with its activation being orchestrated through the assembly and activation of the PANoptosome complex. As central hubs for both cellular energy metabolism and death signaling, mitochondria play a pivotal role in this process by functionally interacting with four major PANoptotic sensors—ZBP1, AIM2, RIPK1, and NLRP12. These interactions coordinate cell fate decisions through a sophisticated multilayered regulatory mechanism that modulates cross-talk among the three cell death pathways (Fig. 4).

### Co-regulatory mechanisms of mitochondria and pan-apoptotic vesicles

#### Mitochondrial DNA release and recognition

Mitochondrial dysfunction, including the opening of mPTP and oxidative stress, results in the leakage of mtDNA into the cytoplasm. This activates DNA sensors such as ZBP1, AIM2, and cGAS, triggering inflammatory signaling pathways and promoting PANoptosome assembly. Oxidized mtDNA can form Z-DNA structures, which are recognized by ZBP1, recruiting RIPK1 and NLRP3 to form a functional complex [50]. Meanwhile, AIM2 binds cytoplasmic double-stranded DNA via its HIN domain, driving the integration of inflammasomes into the PANoptosome [51]. Additionally, mtDNA activates type I interferon signaling through the cGAS-STING pathway and upregulates ZBP1 expression, indirectly enhancing PANoptosis signaling [50].

#### Cascade amplification of ROS

Mitochondria are the main source of ROS. mtROS enhances its kinase activity by oxidatively modifying key molecules and promotes PANoptosome assembly [52]. ROS activate cysteine residues of NLRP12 and stabilize its interaction with ASC [53]. ROS activate the NLRP3 inflammasome through a positive feedback loop that amplifies ZBP1-PANoptosome signaling [52].

#### Regulation of mitochondrial dynamics

The balance between mitochondrial fission and fusion dynamics directly affects PANoptosome activity: drp1-mediated mitochondrial fission promotes Bax/Bak oligomerization and cytochrome c release, which synergistically enhances death signaling with RIPK1 [52]. Whereas, Mfn1/2-mediated fusion suppresses mtDNA leakage through maintenance of membrane integrity, retarding the NLRP12-PANoptosome activation [53].

#### Regulation of mitochondrial dynamics

Mitochondrial energy metabolism regulates lipid droplet-mitochondrial interactions via the AMPK-Rab8a axis, which indirectly modulates RIPK1-PANoptosome stability [54].

It is important to note that mitochondrial actions on the four pan-apoptotic vesicles do not exist in isolation, but are cross-linked and coordinated, and interact with each other. In addition to the above common regulatory mechanisms of mitochondria on the four pan-apoptotic vesicles, mitochondria also mediate interactions among the four vesicles. mtDNA synergistically activates ZBP1 with AIM2, which regulates the innate immune sensors pyrin and ZBP1 to drive inflammatory signaling and PANoptosis [55]. The death structural domain inhibits ZBP1- and TRIF-mediated cell death and inflammation [56]. Four pan-apoptotic vesicles often interact, and when mitochondrial damage triggers vesicle interactions, they amplify death signals and exacerbate mitochondrial collapse [57].

### Individual properties of PANoptosomes

ZBP1 specifically recognizes Z-DNA through its Zα domain and relies on the RHIM domain to bind RIPK1 and RIPK3, forming filamentous structures that activate MAVS signaling on the mitochondrial surface [58]. In antiviral immunity, ZBP1 directly binds viral Z-RNA, inducing necroptosis to restrict tumor growth [59]. Additionally, it exerts cardioprotective effects in cardiomyocytes by suppressing mtDNA-driven inflammation [60].

AIM2 functions as a cytoplasmic dsDNA sensor that recognizes pathogenic or host-derived aberrant DNA through its HIN domain, subsequently recruiting Pyrin and ZBP1 to form an inflammasome complex [55]. Emerging evidence demonstrates its critical role in various pathological conditions: in cerebral hemorrhage models, cytoplasmic mtDNA activates the AIM2 inflammasome and exacerbates neuronal apoptosis, while in non-alcoholic fatty liver disease, increased mtDNA synthesis drives AIM2 inflammasome activation and promotes hepatocyte pyroptosis [61, 62].

RIPK1 regulates PANoptosome activation through site-specific phosphorylation: S166 autophosphorylation promotes its pro-death function, while S321 phosphorylation exerts an inhibitory effect [63]. The kinase activity of RIPK1 is further enhanced through interaction with ZBP1, amplifying PANoptosome assembly [50, 52].

NLRP12 plays a protective role in mitochondrial homeostasis by suppressing inflammatory factor release through negative regulation of the NF-κB pathway and mediating IRF1-dependent responses to stimuli such as heme [64, 65]. Additionally, mitochondrial ROS facilitate the integration of inflammasomes with the PANoptosome by promoting NLRP12-ASC interactions through oxidative modifications [53].

### Cross-regulation of PANoptosis with other cell death modalities

In addition to apoptosis, necrosis, and pyroptosis, mitochondria are involved in various cell death pathways [66]. PANoptosis may interact with these pathways or act as a downstream effect. Mitochondria, as signal integration centers, regulate cell death from transitions and interactions by modulating ROS, mtDNA release, and metabolic status to determine the ultimate cell fate [67]. An in-depth study of the molecular crossover of mitochondria between PANoptosis and other forms of death will help to clarify their dominant role in disease and therapeutic potential.

## Role of mitochondria in various diseases

**Fig. 5:**
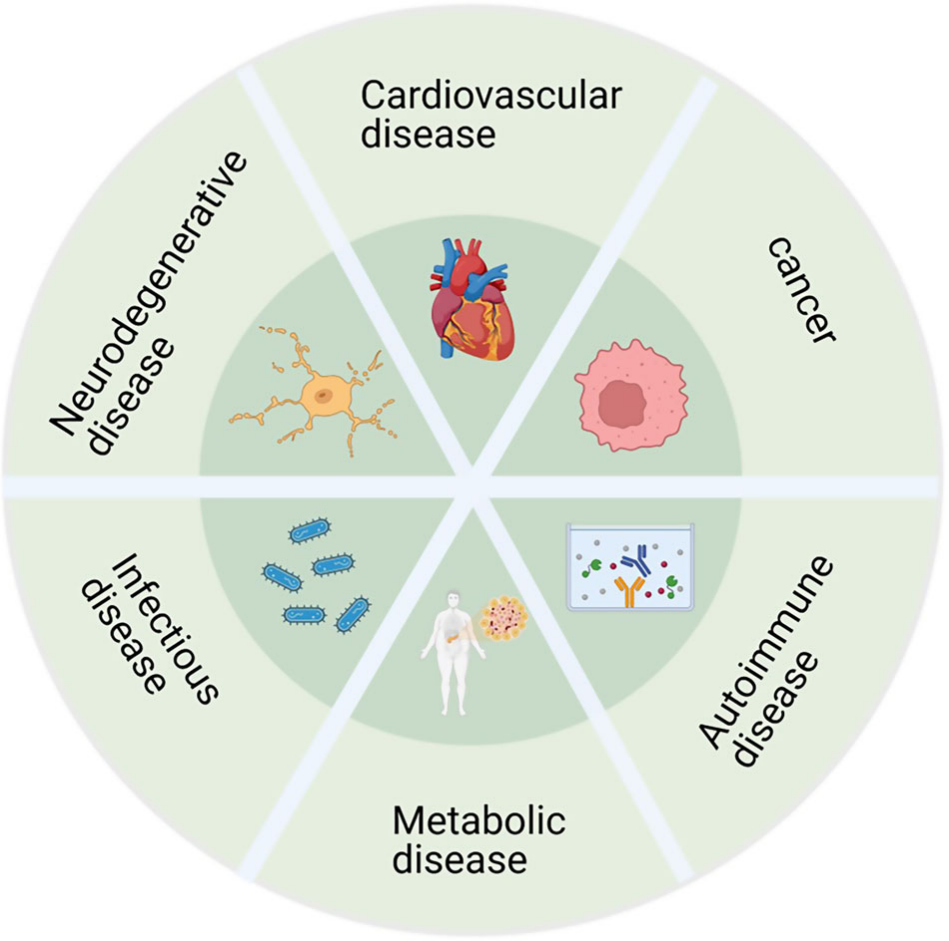
The role of mitochondria in disease. Mitochondria play a role in a variety of diseases, including but not limited to neurodegenerative, cardiovascular, cancer, autoimmune, metabolic, and infectious diseases.

Mitochondria serve as the master regulatory platform of the intrinsic apoptosis pathway, playing a central role in cellular fate determination. By modulating MOMP, releasing Cyt c, mtROS, and mtDNA, they integrate metabolic, oxidative stress, and inflammatory signals to trigger the caspase-9/3 cascade, thereby executing cellular clearance. Apoptosis not only constitutes a fundamental module of PANoptosis but also provides disease-specific therapeutic targets due to its mitochondria-dependent nature. In recent years, precision therapeutic strategies targeting the mitochondrial apoptosis pathway have achieved significant progress in various diseases, demonstrating broad clinical translation potential [68] (Fig. 5).

### Role of mitochondria in neurodegenerative diseases

Neurons, as cell types highly dependent on mitochondrial energy supply and homeostasis regulation, are exquisitely sensitive to mitochondrial dysfunction. In Alzheimer’s disease and Parkinson’s disease, the accumulation of mtROS, aberrant opening of the mPTP, and dysregulation of calcium (Ca²⁺) homeostasis can activate the p53-BAX pathway, triggering BAX/BAK-dependent MOMP. This process facilitates the release of pro-apoptotic factors such as Cyt c and second mitochondria-derived activator of caspases, initiating the caspase-9/3 cascade and ultimately leading to irreversible neuronal apoptosis [69, 70]. Moreover, misfolded proteins like amyloid-beta (Aβ) and alpha-synuclein (α-syn) exacerbate membrane destabilization and amplify apoptotic signaling by promoting BAX oligomerization on the mitochondrial outer membrane [71]. Targeting these pathological mechanisms, mitochondria-directed therapeutics have shown promise: the antioxidant MitoQ significantly reduces mtROS levels, inhibits BAX translocation, and suppresses dynamin-related protein 1 (Drp1)-mediated mitochondrial fission, thereby protecting neurons from premature apoptosis, while the cardiolipin stabilizer SS-31 prevents Cyt c release and loss of mitochondrial membrane potential, demonstrating robust neuroprotection in models of subarachnoid hemorrhage [72, 73].

### Role of mitochondria in cardiovascular diseases

In myocardial ischemia-reperfusion injury, mitochondria serve as the central hub of stress response and are prone to pathological opening of mPTP, leading to membrane potential collapse and subsequent release of Cyt c and SMAC, which rapidly activates the caspase-dependent apoptotic pathway [74]. Concurrently, leaked mtDNA can trigger the TLR9-p38 MAPK-NF-κB axis, amplifying inflammatory responses and exacerbating cardiomyocyte death [75]. In the context of chronic heart failure, lipid metabolism disorders further promote apoptosis through activation of the BIM-BAX pathway [76]. Therapeutically, the TSPO ligand TRO40303 has been shown to delay mPTP opening timing, significantly reduce infarct size, and slow apoptotic progression [77]. Meanwhile, MitoTEMPO, as a ROS scavenger, not only maintains electron transport chain function but also reduces mtROS-induced MOMP occurrence [78]. These interventions provide effective strategies targeting mitochondrial stability, offering promising prospects for protective treatment of cardiovascular events.

### Role of mitochondria in cancer

Cancer cells frequently evade mitochondria-mediated apoptosis through multiple mechanisms, including Warburg effect-induced suppression of oxidative phosphorylation, decreased membrane potential, overexpression of anti-apoptotic BCL-2 family proteins (BCL-2, BCL-xL, MCL-1), and loss of p53 function, all of which can block BAX/BAK activation and prevent MOMP [79]. Consequently, overcoming mitochondrial apoptosis resistance has emerged as a crucial strategy for reversing tumor drug resistance and inducing cell death. The BCL-2-targeting drug Venetoclax restores pro-apoptotic signaling by dissociating BCL-2/BIM complexes, demonstrating a 79% objective response rate in chronic lymphocytic leukemia patients [80]. Next-generation inhibitors, including the MCL-1 inhibitor S63845 and BCL-xL inhibitor A-1331852, effectively induce BAX-dependent mitochondrial apoptosis across various solid tumors, significantly enhancing tumor sensitivity to radiotherapy and chemotherapy [81, 82]. Additionally, the p53 conformation-correcting agent APR-246 reactivates BAX transcription, triggering MOMP and cell death, with clinical efficacy validated in myelodysplastic syndromes [83].

### Role of mitochondria in metabolic diseases

Under metabolic disorders, particularly hyperglycemic and hyperlipidemic conditions, pancreatic β-cells are prone to mitochondrial stress. Elevated mtROS activates the UCP2 proton channel, reducing mitochondrial membrane potential (ΔΨm) and inhibiting ATP synthesis. Subsequent ATP depletion triggers the JNK pathway, leading to BIM-BAX-dependent MOMP and apoptotic signaling [84]. Additionally, ROS oxidizes PTEN and activates AKT, which suppresses FOXO1-mediated BIM transcription, establishing a pathological cycle of apoptosis inhibition and insulin resistance [85]. The classical metabolic regulator metformin exhibits dual protective effects: it not only inhibits mitochondrial complex I and activates the AMPK-BIM axis to eliminate dysfunctional cells, but also reduces mtROS levels through SIRT3 signaling, thereby improving peripheral tissue insulin sensitivity and cellular stress responses [84, 85]. These dual functions in apoptosis regulation and metabolic intervention make metformin a unique therapeutic agent in diabetes treatment, combining both cytoprotective and clearance effects.

### Role of mitochondria in autoimmune diseases

In systemic lupus erythematosus, dysregulated mitochondrial apoptosis is closely associated with immune tolerance breakdown. Aberrant activation of the FASL-caspase-8 axis triggers massive apoptosis of T/B lymphocytes, releasing apoptosomes and mtDNA that activate the cGAS-STING pathway and amplify type I interferon responses [86]. Concurrent impairment of mitophagy exacerbates mtDNA leakage and inflammatory pathway activation. To target these pathological mechanisms, the BAK inhibitor BKI-87C effectively blocks MOMP formation, reducing Cyt c and mtDNA release while decreasing T-cell apoptosis and immune activation levels [87]. Meanwhile, DNase I nanoparticles demonstrate therapeutic efficacy by degrading extracellular mtDNA, achieving a 60% reduction in anti-dsDNA antibody titers in SLE mouse models, thereby validating the therapeutic potential of targeting the mtDNA-apoptosis interconnected pathway [86].

### Role of mitochondria in infectious diseases

During viral infection, host cells can activate mitochondrial apoptosis through the MAVS signaling platform to restrict pathogen replication. The formation of MOMP, Cyt c release, and caspase-9/3 activation constitute the primary mechanism for eliminating infected cells. However, pathogens such as influenza virus and HBV can evade apoptotic clearance by suppressing mitochondrial membrane potential and caspase activity [88].

The mitochondria-targeted copper ionophore elesclomol represents a novel therapeutic approach by inducing iron-sulfur cluster disruption and ROS burst, triggering MOMP formation and caspase-dependent apoptosis. In HBV-infected hepatocytes, elesclomol demonstrates approximately 90% selective cytotoxicity while showing minimal toxicity toward normal liver cells [89]. This strategy exemplifies an innovative “enhanced mitochondrial apoptosis” paradigm for antiviral therapy, characterized by high target specificity and promising clinical potential.

## The bridging role of mitochondria in drug action and disease intervention

### Mitochondria in Bcl-2 family—related drug mechanisms

BCL-2 family proteins act as core regulators of the mitochondrial apoptotic pathway, determining cell fate through a dynamic balance between pro- and anti-apoptotic members [90]. Upon activation of pro-apoptotic proteins, Bax and Bak oligomerize at the mitochondrial outer membrane to form a pore, triggering an increase in the permeability of MOMP, which not only releases cytochrome c to activate Caspase-9/3-mediated apoptosis, but also releases mtDNA and ROS, triggering necrotic apoptosis mediated by RIPK3-MLKL and focalization by the NLRP3-ASC- Caspase-1 pathway, forming a “three-way” mechanism of PANoptosis. Anti-apoptotic proteins, on the other hand, inhibit MOMP and pan-apoptosis initiation by binding to Bax/Bak or sequestering BH3-only proteins to maintain cell survival.

Based on the critical regulation of PANoptosis by the BCL-2 family, strategies targeting anti-apoptotic proteins show great promise. The highly selective BCL-2 inhibitor Venetoclax (ABT-199) achieved breakthrough efficacy in chronic lymphocytic leukemia (CLL) by deregulating Bax/Bak and activating the mitochondrial apoptotic pathway [91]. Navitoclax (ABT-263) inhibited both BCL-2 and BCL-xL in lymphoma and small cell lung cancer [92, 93]. The next-generation MCL-1 inhibitor S63845 and the highly selective BCL-xL inhibitor A-1331852 regulate tumor mitochondrial membrane stability by precisely targeting different anti-apoptotic isoforms, providing a new direction for PANoptosis induction in solid tumors [82].

### Mitochondria in mPTP-related drug mechanisms

The mitochondrial permeability transition pore is a non-selective macromolecular channel located between the inner and outer membranes, and its degree of opening directly affects the stability of the mitochondrial membrane potential and cell fate. mPTP over-opening leads to the collapse of the mitochondrial membrane potential, ATP depletion, and release of cytochrome c, which activates mitochondria-mediated apoptotic pathways, and then induces multiple modes of cell death, including apoptosis, necrosis, and pyroptosis in PANoptosis. mPTP over-opening results in the initiation of various cell death modes. The transient opening of mPTP plays an adaptive role in regulating intracellular calcium homeostasis, ROS signaling, and metabolic homeostasis to support cellular stress response and survival. However, tumor cells reduce the frequency of mPTP opening by modulating mPTP components, such as inhibiting CypD expression or enhancing anti-apoptotic protein activity, which blocks PANoptosis and promotes tumor progression and chemoresistance. Pharmacologically, cyclosporin A blocks CypD interaction with adenine nucleotide transporter, inhibits mPTP over-opening, and protects tissues from pan-apoptosis associated with ischemia-reperfusion injury [94].

### Mitochondria in ROS-related drug mechanisms

Mitochondria are the main source of ROS, and moderate ROS are involved in regulating signals such as NF-κB and HIF-1α to promote cell proliferation and immune regulation. Excessive ROS triggers oxidative stress, damages mtDNA, inhibits the electron transport chain, leads to impaired ATP synthesis, activates the mitochondrial apoptotic pathway, and promotes pan-apoptosis. ROS overload also promotes the assembly of the PANoptosome, which synergistically induces apoptosis, necrosis, and pyroptosis. It was found that NFS1 deficiency enhances tumor cell sensitivity to the chemotherapeutic agent oxaliplatin, triggering PANoptosis by elevating ROS levels [95]. This targets PDK1, which disrupts glucose metabolism, induces a ROS burst, inhibits the PI3K/AKT/mTOR pathway, activates Caspase-3/9-dependent apoptosis, and promotes pan-apoptosis [96]. Anticancer drugs utilize the strategy of inducing mitochondrial ROS.

### Mitochondria in metabolism-related drug mechanisms

Metabolic disruption is a novel strategy to modulate PANoptosis. The classical drug metformin induces tumor cell death by inhibiting mitochondrial complex I, decreasing cellular energy metabolism, elevating AMP levels, activating AMPK signaling, inhibiting glucagon-induced cAMP synthesis, and deregulating PANoptosome inhibition [97]. NDUFB4 deletion leads to the release of mtROS and mtDNA that Activation of NLRP3 inflammasome and RIPK3 promotes Caspase-9/3-mediated PANoptosis, energetic stress, promotes HMGB1 and ATP efflux, enhances immunogenic cell death, and boosts immunotherapeutic efficacy in small-cell lung cancer and ovarian cancer cellular metabolism and multiple cell death pathways, opening up new directions for cancer therapy [98, 99].

### Mitochondria in antioxidants mechanisms

Mitochondrial redox imbalance plays a central role in the pathogenesis of numerous diseases. Mitochondria-targeted antioxidants demonstrate therapeutic potential through distinct mechanisms: N-acetylcysteine effectively scavenges mtROS, suppresses excessive PANoptosis activation, and preserves myocardial and renal tubular cell function via the SIRT3-SOD2-GPX4 axis [100]. Similarly, α-lipoic acid serves dual roles as both a tricarboxylic acid cycle cofactor for metabolic homeostasis and a pro-oxidant that induces ROS bursts in cancer cells. LA inhibits PI3K-AKT-mTOR signaling and synergizes with 5-fluorouracil to enhance chemosensitivity through PANoptosis activation [101, 102]. Furthermore, mitoQ, as a mitochondria-specific antioxidant, precisely eliminates mtROS and disrupts androgen receptor-NLRP3 inflammasome interactions, thereby ameliorating oxidative stress and attenuating cell proliferation in benign prostatic hyperplasia [103]. Collectively, these findings highlight how targeted modulation of mitochondrial redox homeostasis by antioxidants represents a promising therapeutic strategy for controlling PANoptosis and associated diseases.

### Mitochondria in mTOR inhibitors mechanisms

The mTOR pathway regulates cell growth, proliferation, autophagy, and metabolism and is a signaling hub for multiple cell fate decisions. Rapamycin inhibits mTORC1 by binding to FKBP12, decreasing the activation of intracellular Caspase-3, Caspase-9, and RIPK1/RIPK3, and inhibiting multiple cell death pathways associated with PANoptosis [104]. In addition, mTOR inhibition promotes autophagy, participates in mitochondrial quality control, attenuates mitochondrial stress, and synergistically regulates the balance between cell death and survival (Table 1).

**Table 1:** Mechanisms and disease applications of drugs targeting mitochondrial pathways.

Types	Drugs	Mechanism	Related applications	Reference
Bcl-2 protein family-related drug	ABT-199	Inhibition of Bcl-2 promoted cell apoptosis	Acute myeloid leukemia	[105]
	ABT-737	Inhibition of Bcl-2 and Bcl-xL promoted cell apoptosis	Breast cancer	[106]
			small cell lung cancer	[107]
	Navitoclax	Inhibition of Bcl-2 promoted cell apoptosis	Acute lymphoblastic leukemia	[108]
	S63845	Activation of Bax/Bak-dependent mitochondrial apoptosis pathway inhibits McL1-dependent cancer cells	Multiple myeloma	[109]
			Breast cancer	[110]
	A-1331852	Inhibition of Bcl-xL promoted cell death	Acute myeloid leukemia	[111]
mPTP-related drug	Cyclosporin A	Chelate-free Ca^2+^ to delay mPTP opening	Ischemia-reperfusion injury	[11]
ROS-related drug	δ-TT	Induce mitochondrial dysfunction and promote ROS production	Melanoma	[112]
	AAZ2	Promote ROS production and induce mitochondria-dependent apoptosis	Gastric cancer	[113]
			Osteosarcoma	[114]
Metabolism-related drug	Metformin	Inhibition of mitochondrial complex I activates the AMPK/mTOR pathway	diabetes	[115]
			Ovarian cancer	[116]
			Small-cell lung cancer	[117]
Antioxidants	NAC	Scavenging ROS and maintaining mitochondrial REDOX homeostasis	Diabetic nephropathy	[118, 119]
	CoQ10	Maintain mitochondrial membrane potential and remove free radicals	Heart failure	[120]
	LA	Clear ROS and promote the production of reducing Ca²⁺	Colorectal cancer	[121]
	MitoQ	Target mitochondria and reduce ROS	Prostate cancer	[122]
mTOR inhibitors	Rapamycin	Inhibition of mTOR pathway reduces the activation of ROS and Caspase	Head and neck cancer	[123]
			Adenocarcinoma of the lung	[124]

## Conclusion

Mitochondria stand at the crossroads of PANoptosis, serving as both sensors and amplifiers that coordinate apoptosis, necroptosis, and pyroptosis into an integrated cell death response. Their ability to regulate MOMP, release mtDNA, and modulate ROS production positions them as central players in determining cellular fate under stress. The crosstalk between these pathways—such as GSDMD-mediated mitochondrial damage triggering MOMP, or RIPK3-driven metabolic rewiring enhancing ROS production—reveals a tightly interconnected network where mitochondrial dysfunction can tip the balance toward inflammatory or immunologically silent cell death.

The implications for disease are profound. In neurodegeneration, persistent mitochondrial stress may drive PANoptotic neuronal loss, while in cancer, metabolic adaptations often suppress mitochondrial death signaling to promote survival. Therapeutic strategies targeting these mechanisms—such as BCL-2 inhibitors to restore apoptosis or NLRP3 blockers to limit pyroptosis—must account for the dynamic interplay between these pathways. However, challenges remain, including tissue-specific variations in mitochondrial susceptibility to PANoptosis and the risk of off-target effects when modulating fundamental processes like ROS signaling.

Looking ahead, key questions center on how mitochondrial dynamics influence PANoptosome assembly and whether organelle-specific interventions can selectively modulate cell death in disease contexts. Advances in mitochondrial proteomics and real-time imaging will be critical in dissecting these mechanisms. By refining our understanding of mitochondrial control over PANoptosis, we can develop more precise therapies for conditions where dysregulated cell death fuels pathology.
